# Combining Pericapsular Nerve Group (PENG) Block With the Supra-Inguinal Fascia Iliaca Block (SIFICB) for Perioperative Analgesia and Functional Recovery in Patients Undergoing Hip Surgeries: A Retrospective Case Series

**DOI:** 10.7759/cureus.36374

**Published:** 2023-03-19

**Authors:** Devyani J Desai, Neha Shah, Pinal Bumiya

**Affiliations:** 1 Anesthesiology, Medical College, Sir Sayajirao General Hospital, Baroda, IND

**Keywords:** perioperative analgesia, ultrasound guided, nerve blocks, functional recovery, trauma, hip surgery, inter-fascial plane block, fascia iliaca compartment block (ficb), peng block

## Abstract

Background: The complex innervation of the hip joint may require a combined peripheral nerve block technique for perioperative effective analgesia and early recovery. The pericapsular nerve group (PENG) and suprainguinal fascia iliaca compartment blocks (SIFICB) are interfascial plane blocks aiming to involve the femoral, obturator, accessory obturator, and lateral femoral cutaneous nerves. The data still lacks in providing the standard of care for patients undergoing hip surgery. In this case series, we studied the efficacy of ultrasound-guided combined PENG block and SIFICB for perioperative analgesia and functional recovery in patients posted for hip surgery.

Method: We studied 10 adults of either gender who underwent close reduction and internal fixation of hip fracture. Before receiving spinal anesthesia, all patients had PENG block and SIFICB with 10 ml and 20 ml of local anesthetics respectively. Patients were observed for ease of giving sitting position for spinal anesthesia (EOSP), visual analogue score (VAS) at rest and 15° leg elevation, duration of postoperative analgesia, the cumulative requirement of rescue analgesic at 48 hours and ability of patients to undergo weight-bearing trial postoperatively.

Result: The static and dynamic VAS before receiving spinal anesthesia and postoperatively, was reduced compared to pre-block. The optimal position for delivering spinal anesthesia was possible to achieve as the patients were able to sit comfortably after 10 minutes of receiving both blocks. Duration of postoperative analgesia also extended up to 18 hours with the cumulative requirement of injection tramadol restricted to two doses postoperatively. All were able to walk down a minimum of 55 steps after 48 hours of completion of surgery.

Conclusion: Combining PENG block along with SIFICB is effective in the provision of perioperative analgesia with a considerable reduction in opioids and enhanced functional recovery due to motor sparing effect after surgical repair of the hip fracture.

## Introduction

With the projected incidence to reach about 6.3 million by 2050, hip fractures are the most frequently encountered fragility fractures globally [1], being common in the elderly with co-morbidities resulting in hemodynamic instability and increased morbidity and mortality. Also, associated with severe pain, continuum analgesia is a mainstay in the pre-hospital, preoperative and post-operative periods till the phase of final rehabilitation for optimal care of patients with hip fractures [2,3]. Having complex innervation, the pain following fractures or surgeries involving the hip joint is difficult to get rid of and may lead to serious implications-i.e., cognitive decline, delirium, and depression. Delayed mobilization and functional recovery followed after incomplete analgesia, leading to complications such as atelectasis, pneumonia, deep vein thrombosis, pulmonary embolism, increased chances of myocardial infarction due to sympathetic stress response, etc. [4]. The established practice to decrease pain using opioids in hip fracture surgery may result in compromised outcomes in the frail and elderly population with nausea-vomiting, constipation, delirium, and respiratory depression. This popularized the regional analgesic techniques in the surgical treatment of hip fractures. Femoral nerve (FN) and fascia iliaca compartment (FICB) block are documented to provide good peri-operative analgesia with reduced need for opioids [4-7]. The latest, pericapsular nerve group (PENG) block, is an interfacial plane block targeting the articular branches of the femoral, obturator (ON), and accessory obturator nerves (AON) at the hip [8]. An ability to perform in supine positioning, which is especially important in patients with acute hip fractures or chronic pain is an indigenous advantage of PENG block. Due to the blockade of only sensory articular branches, substantial motor weakness is unexpected. But exclusive use of PENG block for analgesia in hip fracture patients is not sufficient as it doesn't involve the cutaneous pain-generating area supplied by the lateral femoral cutaneous nerve (LFCN) [9]. Many options are explored for providing effective analgesia for patients undergoing hip surgeries. We studied a combination of PENG block with supra-inguinal fascia iliaca block (SIFICB) in patients with hip fractures and observed their efficacy related to perioperative analgesia and functional recovery.

## Materials and methods

This case series included 10 adult patients of an equivalent age (55-68 years old of either gender) who underwent close reduction and internal fixation of hip fracture at a tertiary care center and provided written consent for the nerve blocks. On arrival at the operation theatre, a multipara monitor for non-invasive blood pressure (NIBP), continuous electrocardiogram (ECG), and pulse oximeter (SPO2) were attached. The preoperative visual analogue score (VAS) was noted. Under all aseptic precautions, all patients received ultrasound-guided (USG) PENG block and SIFICB before spinal anesthesia, in a supine position as described below. The total drug volume made was 30 ml with 0.25% bupivacaine and 8mg dexamethasone (15 ml of 0.5% bupivacaine + 13 ml sterile water + 2 ml dexamethasone = total 30 ml).

Technique of PENG block

**Figure 1:**
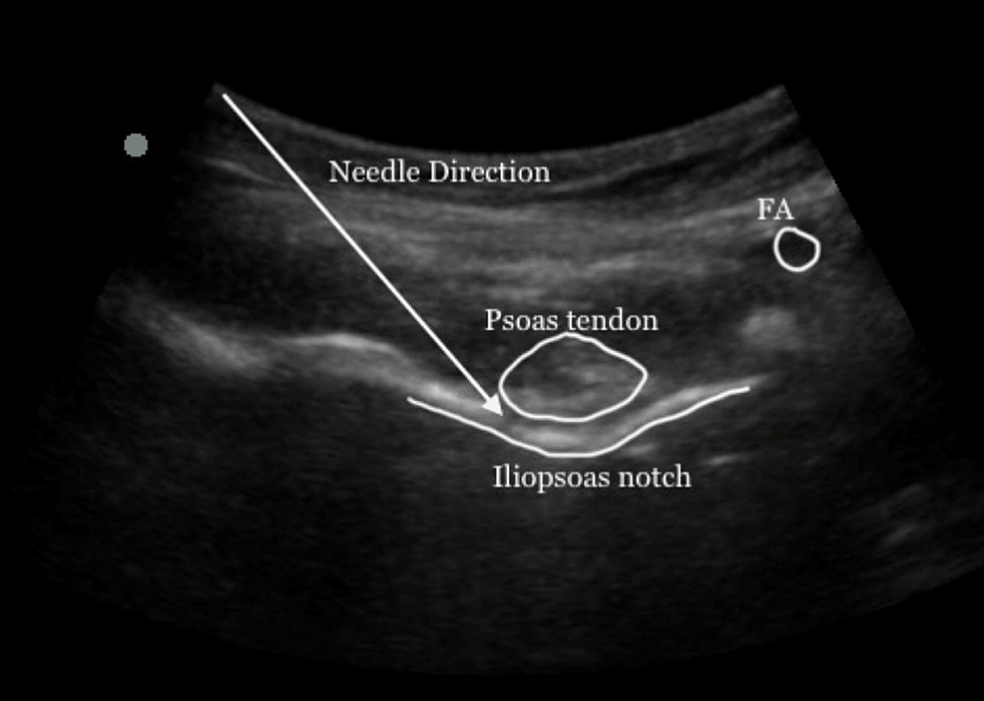
Ultrasound image of PENG block. The lateromedial approach of PENG block. The white arrow shows the direction of the needle in the plane between the psoas tendon and periosteum and between the anterior inferior iliac spine and iliopubic eminence or iliopsoas notch. FA: femoral artery, PENG: pericapsular nerve group

A low-frequency (2-5 MHz) curvilinear transducer was positioned horizontally over the anterior inferior iliac spine (AIIS). The pubic ramus, ilio-pubic eminence, and femoral artery were identified by sliding over the probe inferiorly. Then the probe was rotated clockwise or counterclockwise approximately 45 degrees to align with the pubic ramus [4]. Using the in-plane technique, the 22G, 10 cm long, echogenic needle was introduced by keeping the direction lateral to medial in the musculofascial plane between the psoas tendon and the pubic ramus. The accurate position of the needle was confirmed by hydro dissection and spread under the illo-psoas muscle. Then 10 cc of the drug was injected (Figure 1).

Technique of SIFICB

**Figure 2:**
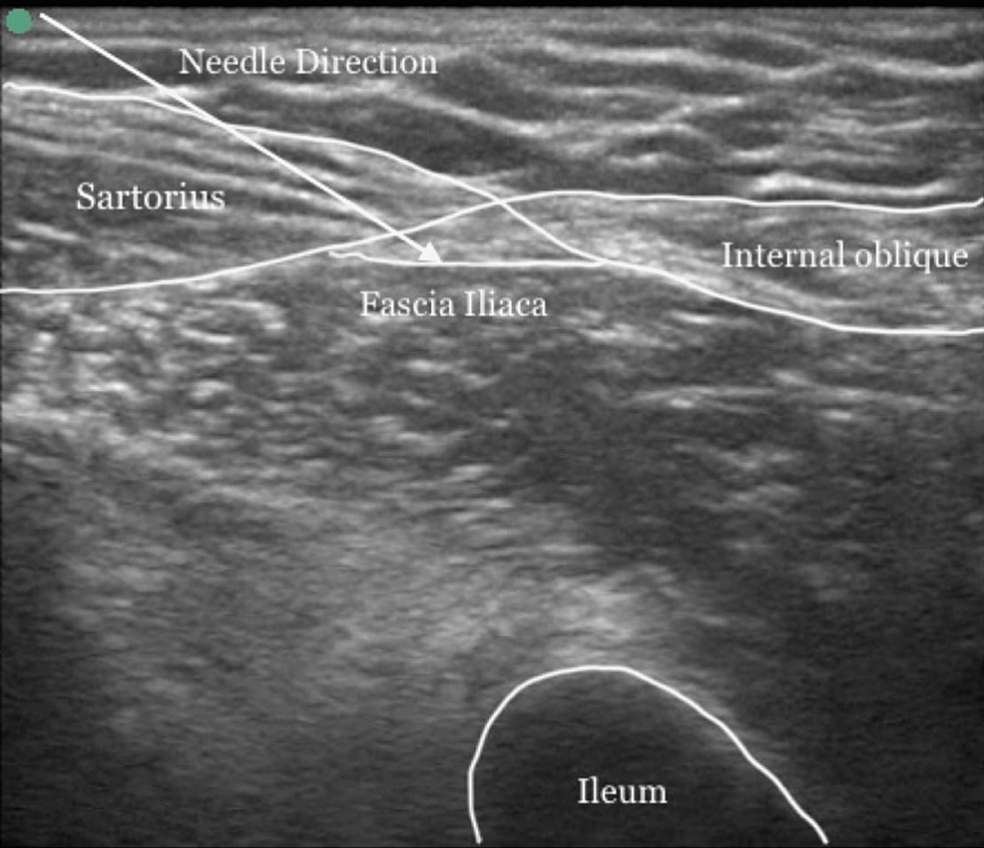
Ultrasound image of SIFICB. The appearance of the "BOW-TIE" sign formed by the sartorius and internal oblique muscles. The white arrow shows the direction of the needle to pierce the fascia iliaca. SIFICB: suprainguinal fascia iliaca compartment block

A high-frequency linear transducer (8-14 MHz) probe was kept longitudinally over the inguinal crease to locate the anterior superior iliac spine, and then to glide medially. The “bow-tie sign” formed by the internal oblique and the sartorius muscles, the iliopsoas muscle and fascia iliaca were identified [10]. A 22G, 5cm long, echogenic needle was inserted 1 cm above the inguinal ligament. With the in-plane technique, the tip of the needle is positioned underneath the fascia iliaca, lateral to the femoral artery, and confirmed with the separation of the iliacus muscle by hydro dissection. Then the needle was advanced cranially while continuing with the hydro dissection. The injection was accomplished once the spread of local anesthetic was observed in the cephalic direction from where the iliacus muscle passes under the abdominal muscles. A total of 20 cc drug was injected (Figure 2).

The VAS at rest and 15° leg elevation were measured 10 min later after the completion of both blocks. The ease of giving a sitting position for spinal anesthesia (EOSP) was assessed by the EOSP score as 0-Not satisfactory, 1-Satisfactory, 2-Good, and 3-Optimal [11]. After 10 minutes, spinal anesthesia was performed using a 2.5 ml injection of bupivacaine 0.5% in L3-4 space under all sterile precautions. Intraoperative fluids and blood were replaced according to the requirement. Postoperatively, all patients were shifted to the post anaesthesia care unit (PACU) and received an intravenous injection of paracetamol 1gm and then twice a day. The static and dynamic VAS for analgesia in the PACU as well as at six, 12, 18, 24, and 48 hours after surgery were obtained. When VAS >/=4, patients received injection tramadol 2mg/kg intravenously and the requirement of cumulative dosage of injection tramadol was obtained for the first 48 hours postoperatively. Peri-operatively, all the patients were observed for hemodynamic parameters, the static, and dynamic VAS, duration of analgesia (time from the end point of giving both blocks till the patient received the first dose of rescue analgesic, injection tramadol) and the ability of patients to undergo weight-bearing trial at the 24 hours and walk with support after 48 hours. All patients were asked to carry out quadriceps exercises as instructed and guided by the physiotherapist.

## Results

All patients underwent close reduction and internal fixation of hip fractures with a surgical duration of 3.5-5 hours (Table 1). We noticed reduced static and dynamic VAS at 10 min, six, 12, 18, 24, and 48 hours respectively as compared to pre-block which was 8 (7-9) and 9 (8-10) respectively. The optimal position for delivering spinal anesthesia was possible to achieve (EOSP score 3) as the patients were able to sit comfortably after 10 minutes of receiving both blocks due to reduced VAS. Duration of postoperative analgesia also extended up to 18 hours (six to 24 hours) with the cumulative requirement of injection tramadol restricted to two doses postoperatively suggesting that dual blocks provided effective pain relief after the hip surgery. All patients were able to do 15-degree elevation at the hip joint actively within six hours which indicated good pain relief as well as less motor blockade. The patient’s cooperation during physiotherapy was also good; all could stand with the support when weight bearing trial was given after 24 hours postoperatively. All were able to walk down a minimum of 55 steps after 48 hours of completion of surgery. We did not encounter any complications like local anesthetic toxicity, falls due to muscle weakness, or nausea/vomiting (Table 2).

**Table 1:** Demographic data (median and range)

Age	60 (55-68) years
Gender (M:F)	4:6
Duration of surgery	3.5 - 5 hours
Type of surgery	Close reduction and internal fixation

**Table 2:** EOSP - Ease of giving sitting position for spinal anesthesia, VAS -Visual analogue score, min - minutes, hrs - hours, Duration of analgesia (median and range)

Parameter	Score	
EOSP score	3 (2-3)	
VAS	AT REST	AT 15 DEGREE ELEVATION
PRE-BLOCK	8 (7—9)	9 (8—10)
10 min after block	0 (0—2)	0 (0—2)
6 hrs	0 (0—2)	0 (0—2)
12 hrs	1 (0—3)	2 (0—4)
18 hrs	3 (1—4)	4 (2—4)
24 hrs	4 (2—4)	4 (3—4)
48 hrs	2 (2—4)	4 (3—7)
Duration of analgesia (hours)	18 (16—24)	
Cumulative dosage of rescue analgesic for first 48 hours	2 (1-3)	
Able to walk with support after 48 hours	55 (56-116)	

## Discussion

The combination of PENG block and SIFICB was able to provide effective perioperative analgesia and enhanced functional recovery after a hip surgery. As identified histologically, the anterior and superolateral capsule of the hip joint is rich with nociceptive fibres and the posterior capsule with mechanoreceptors [12]. Being a musculofascial plane block, the PENG is intended to block articular branches of the FN, ON and AON which is necessary for effective analgesia of the hip joint. Blockage of the high branches of the femoral nerve beyond the inguinal ligament which innervates the hip joint is an added advantage associated with this block [13]. It can be performed without taking extra effort as the landmarks like the anterior inferior iliac spine and the iliopubic eminence are easy to identify. The articular branches of the FN, ON and AON are consistently found here. A pooled meta-analysis done by Huda et al. demonstrated complete analgesia with less incidence of motor blockade and better range of motion leading to a shorter time to first walk postoperatively with PENG block in patients with hip arthroplasty [14]. The encouraging results were found with PENG block over other regional blocks as well such as femoral nerve block or fascia iliaca compartment block with no differences in static and dynamic pain scores as well as cumulative opioid consumption during 48 hours with preservation of quadriceps muscle strength after total hip arthroplasty [15]. As it is relatively a new block since 2018, sufficient data with respect to its superiority to other blocks are still lacking. The exclusive use of PENG block as an anesthetic/analgesic block for hip surgery is the pitfall. Pairing with an LFCN block is necessary to administer analgesia over the lateral part of the thigh which is the incision site for hip surgeries in the majority [4]. This will help to enhance the quality and duration of analgesia as observed by Jadon et al. and Roy et al. [16,17]. Many authors have used PENG block with different volumes of local anesthetics from 10 ml to 30 ml to provide analgesia after hip surgery, which could obscure the discrepancy of their results [14-20]. Reviewing the fluoroscopic and cadaveric study, the spread of 5, 10, 15 and 20 ml dye when injected below the psoas tendon and over the superior pubic ramus, adequate diffusion was found with 10 ml volume as well. At higher ages and with a high degree of osteoarthritis, disruption of the psoas bursa as well as degeneration of the iliofemoral and pubofemoral ligaments is constant, so greater diffusion is expected with the small volume of injectate [21]. This justifies the 10 ml volume for the analgesic potency of the PENG block. Girón-Arango and Jadon et al. used 20 mL of 0.25% bupivacaine with dexamethasone successfully but incidental quadriceps weakness after PENG block with 20 ml and 30 ml drug volume was reported by two studies [8,22,23]. Considering the resting and exercise pain, using 10 ml volume was equally effective as 20 ml of drug volume, Lu et al. recommended using 10 ml while performing the PENG block as they found a reduced incidence of quadriceps motor blockade at six hours [23]. This was consistent with our case study. 

Fascia iliaca compartment block is the most frequent addition in the perioperative pain management of hip fracture patients as it is effective and relatively safe with a reduction of delirium and length of hospital stay [2,7]. Superior analgesia and statistically significant shorter time to execute spinal anesthesia were observed for patients receiving FICB compared with opioids [7,24]. The supra-inguinal approach of FICB provides superior analgesic efficacy and higher cephalad spread of local anesthetic agent as opposed to the infra-inguinal approach with a possibility of obtaining an effective block using a smaller amount as suggested by Yamada et al. [24]. Different drug volumes were used for SIFICB by various authors ranging from 20 ml to 60 ml [13,25]. Yamada et al. documented EV50 and EV95 of 0.25% ropivacaine for SIFICB were 15.01 ml and 26.99 ml respectively to block femoral and LFCN while blockade of all three nerves including ON requires 40 ml [10,24]. High doses of local anesthetics are associated with an increased risk of local anesthetic toxicity. As patients with hip fractures are inclined to be lean, the volume of local anesthetics used for regional blocks is a critical factor [24]. Twenty ml volume is needed to reach the FN and LFCN in a cadaver study as stated by Vermeylen et al. with a cadaver weighing between 55 and 72 kg [26]. Data suggested quadriceps weakness after 30 ml of local anesthetics in SIFICB. while higher incidences of motor weakness are reported with 40 ml and 60 mL of ropivacaine 0.5% respectively [15,25,27]. To avoid any untoward effect, we restricted the drug volume (20 ml) injected for SIFICB to target LFCN. This resulted in absence of quadriceps muscle weakness. Movements were possible at hip and knee joints which were asked the patients to do after six hours. As per our institutional protocol, we allowed patients to stand with support after 24 hours. All were effectively able to do physiotherapy starting after 24 hours and able to undergo weight-bearing trials. When the steps were taken into account, all were able to walk with support for at least 55 steps after 48 hours. All patients were discharged three days after the surgery. 

Hip fracture and its surgical intervention render a pain that is not that simple to control; a strong opioid is needed to provide analgesia. The addition of peripheral nerve blocks are being recommended by the National Institute for Health and Care Excellence (NICE) when paracetamol and opioids are not adequate to provide pain relief which in turn also reduces opioid consumption and its side effects. Therefore, finding the balance in the modality of analgesia plays a key role in the “goldilocks condition” [2]. Huda et al. demonstrated significantly reduced opioid consumption in the first 24 hours after surgery and highly extended duration to the first demand of rescue analgesia with the PENG block usage for patients undergoing hip surgery [14]. Authors have reported reduced requirement of morphine in patients with fractured neck femur 24 hours postoperatively [6,7]. FICB as well as PENG block provided better analgesia as opposed to intravenous use of fentanyl for pre-positioning patients during spinal anesthesia for all types of fractured neck femur [7,11]. Stevens et al. demonstrated a remarkable morphine-sparing effect using SIFICB after total hip arthroplasty [28]. The various combination of nerve blocks like PENG with LFCN or PENG with quadratus lumborum (QL) also resulted in the same [9,16]. This coincided with our case study. 

To date, these two blocks studied individually to provide perioperative analgesia for patients undergoing hip surgeries. To the best of our knowledge, this is the first-ever case series combining the PENG block with SIFICB for perioperative analgesia and functional recovery after hip surgeries. According to magnetic resonance imaging and cadaveric studies, the spread of local anesthetics following FICB cannot consistently block the articular branches of ON and the sparing of LFCN is known with the use of a PENG block [4,13]. Therefore, combining these two blocks (PENG+SIFICB) can supplement each other to produce effective analgesia for patients undergoing hip surgeries. This also can be advantageous in terms of limiting the volume of local anaesthetics to avoid any undesirable effects and preservation of motor function. The decreased opioid consumption was demonstrated only up to 24 hours after receiving the PENG block as suggested by Farag et al. which led them to conclude that the analgesic effect post-PENG block wears off after 24 hours [29]. This may be overcome by adding SIFICB to the PENG block which ultimately becomes helpful to improve the outcome of patients after hip surgery. 

A small sample size carries the limitation of this case series. The sensory dermatomal coverage was also not checked as well as combined nerve blockade technique needs to be assessed for motor blockade more objectively. This is a case series; we suggest further randomised controlled studies in future for a better understanding and usefulness of combining these two regional blocks for patients undergoing hip surgery. 

## Conclusions

The PENG block along with SIFICB provided good perioperative analgesia with a comfortable sitting position to deliver spinal anesthesia as well as lower pain scores with opioid-sparing effect postoperatively. The associated motor-sparing effect led to better functional recovery after the surgical repair of the hip fracture. Both techniques can be easily applied under ultrasound guidance and keeping the supine position. Further research is recommended to establish the safety and efficiency of the PENG block with the combination of other regional blocks for patients undergoing hip surgery.
